# Approach to Trauma During Pregnancy

**DOI:** 10.5812/atr.13556

**Published:** 2013-08-01

**Authors:** Masoumeh Abedzadeh-Kalahroudi

**Affiliations:** 1 Trauma Research Center, Kashan University of Medical Sciences, Kashan, IR Iran

Among different groups of population involved with injuries, trauma during pregnancy is a unique event because two patients, the mother and her fetus, are at risk and need evaluation and management.

Trauma is one of the most common causes of maternal mortality (1). Also fetal mortality rate following trauma is reported from 3% to 38% (2). Approximately 6 – 7% of pregnancies get complicated by traumatic injuries (3).

Two thirds of maternal traumas are due to motor vehicle accidents, followed by many other mechanisms including partner violence, fall, burn, suicide, intoxication, sport and pedestrian injuries (1, 2, 4).

Blunt abdominal trauma is responsible for two thirds of injuries during pregnancy and often results from motor vehicle accidents (4). Gunshot, stab wounds and effort for illegal abortion are the most common causes of penetrating trauma (2, 5). In penetrating trauma, probability of maternal organ damage is about 15 – 40% and fetal injury is about 70% (5).

In evaluation and management of trauma in pregnancy understanding of anatomical and physiological changes of this period is very important (4), because diagnosis of many symptoms and interpretation of physical and laboratory findings may be difficult. A multidisciplinary approach with an expert team including trauma surgeon, gynecologist/obstetrician and neonatologist seems to be mandatory (3).

All patients should be observed in an adequately equipped hospital (4). Since severity of injury cannot precisely predict outcomes for the mother and her offspring, therefore evaluation of all pregnant women after any kind of injury is strongly recommended, even in those with minor injuries. The initial goal is evaluation of maternal injuries and stabilization of her general condition. Resuscitation consists of the basic rules of ventilation, homeostasis and treatment of hypovolemia (5). Then after, obtaining a complete history and performing physical examination focusing on vital signs, central nervous system condition and chest wall and extremities movement should be considered. Paraclinical diagnostic evaluations including radiographic and ultrasonographic studies, if needed, should not be delayed because of the concerns about radiation risk to the fetus. In victims with penetrating injuries, internal organs must be evaluated for the possible injuries (4).

Other protocols are depended on the gestational age, injury severity and obstetrical characteristics of the patients (2). Since abruptio placenta is reported in 30 – 50% of patients with major and 5% of patients with minor injuries (3), an external fetal monitoring must be started immediately after stability of maternal hemodynamic status and continued for at least 24 hours for all patients with gestational age of more than 24 weeks (4, 5).

Penetrating uterine injury in those with gestational age of less than 25 weeks should be treated conservatively, and hysterectomy must be limited only to those cases of maternal hemorrhage or fetal death due to uterine laceration (1).

Fetal-maternal hemorrhage is a major complication reported in 28% of all cases, even those with minor trauma, thus Rh-negative mothers should be closely observed and receive Rhogam (4).

Pregnant women are at higher risk for preterm labour, abruptio placenta and delivery within 48 hours of trauma. Also their fetus is at higher risk of death, respiratory distress syndrome, low birth weight and prematurity. Maternal shock is the most common cause of fetal death and is associated with a fetal mortality rate of 80% (2, 4). Feto-maternal hemorrhage, uterine rupture, pelvic fracture, fetal subdural hemorrhage, hydrocephalus, cerebral palsy and fetal spinal fracture are other reported complications of trauma (2).

In a study in Kashan, the prevalence of trauma during pregnancy was reported 1.1% which is higher than other studies. The most common cause of injury in this study was motor vehicle accidents (6).

Evidence show that use of 3-point seat belts and airbags can reduce the probability of maternal and fetal injury following motor vehicle accident. An appropriate use of seat belts may reduce fetal death up to 50%. In another study only 48.7% of women reported that they get information about seat belts use in their prenatal visits (2). Therefore, women should be advised about the importance of the proper seat belt use, and airbags activation.

Physical violence is another cause of maternal trauma. In a study 50% of the women had experienced partner abuse and domestic violence (7). So, it is wise to screen all pregnant women for domestic violence in prenatal care and provide adequate support for them.

Cesarean section, as a rapid method of termination of pregnancy and delivery of the fetus, has an important role in fetal lifesaving. It can also improve maternal care and resuscitation (2).

Since most of the causes of maternal trauma are the same involving general population, which have high incidence in our country, special training and supporting of pregnant women as well as planning for the improvement of public knowledge on this special issue is very important. At first step, preventing the events and in next, when it happened, dealing appropriately with this critical health problem is necessary.
